# Physical co-presence intensity: Measuring dynamic face-to-face interaction potential in public space using social media check-in records

**DOI:** 10.1371/journal.pone.0212004

**Published:** 2019-02-11

**Authors:** Yao Shen, Kayvan Karimi, Stephen Law, Chen Zhong

**Affiliations:** 1 College of Architecture and Urban Planning, Tongji University, Shanghai, P. R. China; 2 The Bartlett Centre for Advanced Spatial Analysis, University College London, London, United Kingdom; 3 Space Syntax Laboratory, Bartlett School of Architecture, University College London, London, United Kingdom; 4 Alan Turing Institute, London, United Kingdom; 5 Kings College London, Strand, London, United Kingdom; University of Oregon, UNITED STATES

## Abstract

Urban public spaces facilitate social interactions between people, reflecting the shifting functionality of spaces. There is no commonly-held consensus on the quantification methods for the dynamic interplay between spatial geometry, urban movement, and face-to-face encounters. Using anonymized social media check-in records from Shanghai, China, this study proposes pipelines for quantifying physical face-to-face encounter potential patterns through public space networks between local and non-local residents sensed by social media over time from space to space, in which social difference, cognitive cost, and time remoteness are integrated as the physical co-presence intensity index. This illustrates the spatiotemporally different ways in which the built environment binds various groups of space users configurationally via urban streets. The variation in face-to-face interaction patterns captures the fine-resolution patterns of urban flows and a new definition of street hierarchy, illustrating how urban public space systems deliver physical meeting opportunities and shape the spatial rhythms of human behavior from the public to the private. The shifting encounter potentials through streets are recognized as reflections of urban centrality structures with social interactions that are spatiotemporally varying, projected in the configurations of urban forms and functions. The results indicate that the occurrence probability of face-to-face encounters is more geometrically scaled than predicted based on the co-location probability of two people using metric distance alone. By adding temporal and social dimensions to urban morphology studies, and the field of space syntax research in particular, we suggest a new approach of analyzing the temporal urban centrality structures of the physical interaction potentials based on trajectory data, which is sensitive to the transformation of the spatial grid. It sheds light on how to adopt urban design as a social instrument to facilitate the dynamically changing social interaction potential in the new data environment, thereby enhancing spatial functionality and the social well-being.

## Introduction

A city is a complex system for the interactions between humans. Although modern communication technologies have extended human interactions from the physical to the non-physical, the built environment still functions as the primary arena in which people encounter one another, socialize, and build confidence. The geographical landscape can be considered as a generator for the (re)production of the temporal process from place to place [1]. This concept has also been fundamental to architectural research, and to configurational studies in particular, which focuses on the interrelation between spatial configuration and urban performance, intermediated by social interactions. Therefore, an explicit understanding of social interactions could be an invaluable tool to assess the social effectiveness of urban design.

The concept of “co-presence” in space syntax theory has been argued to be a by-product of the organization of the built environment, which often manifests itself in people’s movements, since connections between spaces create the possibility of mutual visibility between people [2]. By interlinking the concepts of co-presence in space syntax and related concepts in sociology, Marcus [3] promoted a concept called “spatial capital,” which reflects the geometric distance that people should overcome to be co-present for unfolding the role of the architectural in shaping social capital in space. These efforts contributed to the knowledge translation between urban design and sociology, but failed to explicitly quantify the temporally shifting face-to-face presence patterns within the geometric context of urban space, thereby constraining the implication of urban design for dynamic social well-being in space.

The recent emergence of location-sensitive mobile technologies allows us to quantify the processes behind human dynamic patterns over a large area with fine spatial and temporal resolutions [4][5]. Previous studies have used social media data to infer patterns of human activities [6–9], predict urban mobility patterns based on individual travel records [10–12], and sense social structures and segregation between people in space [13][14]. These efforts provide insights into diverse aspects of the agglomerative interactions between people in a geographical context from a Euclidean scope with arbitrarily-defined unit setting and metrical distance metrics rather than a topo-geometric perspective using in-use urban element as the basic unit and geometric distance indices, which could not sufficiently address how the physical urban form is shared geometrically by people. Consequently, it limits the social translation of urban design from the built form to its dynamic functionality. A systematic investigation equipped with new approaches and enabled by newly emerging data is required to capture the shifting performance of the spatial configuration on interlinking people in urban spaces. It will enable more precise appraisal of the effect of urban design on people’s quality of life in and through public spaces.

From an urban design perspective, the main scope of this article is the delivery of a framework for quantifying the spatiotemporally changing network-based centrality structures of cities, characterized by their shifting roles of physically connecting local and non-local residents, which are sensed by social media check-in records. This focus is multi-fold. First, it introduces a series of measures to quantify the essential aspects of *physical co-presence intensity* (the likelihood that different groups of people encounter each other in the same street at the same time). We extract travel diaries of social media users from their geo-referenced check-ins. People are then characterized as “locals” or “non-locals” for every space they visit during different time periods, depending on the frequency and duration with which they used that space. This classification is essential for architectural research and urban studies which aim to clarify the social embodiment of spatial publicness for people’s on-site interactions [15]. We then provide an example of how this framework can be applied to reflect the social potential of every street, using data from Central Shanghai. Our analysis demonstrates hourly changing centrality structures in a typical working day, as illustrated by the dynamic variation in the patterns of physical co-presence intensity. This is then validated using a survey (“gate count”) of people’s movements in the city to test if the aggregated flows of social media users can represent the global variations of overall pedestrian flows. In the models where other static network centralities are identified as regressors, the physical co-presence intensity measures reasonably classify temporal interactions between urban form and function from day to night. Using a clustering algorithm, we further classify the typology of streets according to the modes of people’s face-to-face interactions. Our results provide a sociological understanding of the street hierarchy system. Finally, this article summarizes the findings and discusses the significance of applying the delivered methods for the advancement of knowledge regarding the dynamics of urban morphologies and their social translation over time.

## Background

Face-to-face co-presence is one of the essential conditions for the occurrence of social interaction [16]. There are a variety of definitions of co-presence, depending on the field, methodology, and spatial scale in question. It can also be defined as a sense of co-existence between people in actual space, or through their virtual communications via various sharing behaviors, such as photo-sharing, “tagging,” or other social media interactions [17]. It has been argued that such “virtual” co-presence can serve as a substitute for face-to-face interactions in various ways, exerting different influences on each another [18][19]. Small-world social networks generally require physical interaction, and therefore urban spaces perform vital roles in the formation and maintenance of social structures [20][21]. The concept of physical co-presence potential has therefore been adopted in the assessment of architectural design, and the “social” performance of cities. The occurrence probability of face-to-face encounters in urban spaces has been related to many aspects of urban vitality, and exploited to measure the publicness of urban spaces [22][23]. At the geographical scale, spatial co-presence potential can be understood through the index of exposure or its mirror-isolation, which describes the inter-group interaction potential and within-group interaction potential in people’s daily activity spaces [24]. Another recent attempt was using joint accessibility, which is the intersection of space-time prisms, to estimate the physical interaction potential by taking into account mobility patterns [25]. These efforts, even though they were conducted on different scales with various approaches, suggested that physical co-presence patterns are a key focus for uncovering the multi-scalar implications of urban design, planning, and other spatial developments, even though our social connections are not currently geographically limited [26].

Physical co-presence potential is rooted in mobility patterns indicating that social interaction is associated with people’s travel choices. This idea has been widely accepted in transport geography with a focus on the collectivity of many individual trips [27][28], which has been validated by studies based on ubiquitous mobility data. Some attempts based on human contact networks have been made [29][30]. With the help of Bluetooth sensors, Kostakos et al. [31] captured the spatiotemporal patterns of mobility, presence, and encounters in spaces around the sensors by tracking the unique identification of each cell phone. Sun et al. [32] studied repeated encounters on public transport using time-resolved social encounter networks extracted from smart-card data. There has also been a focus on exploring the relationship between telecommunications and co-location (the prelude of face-to-face meetings), and it has been suggested that telecommunications strength helps to predict the patterns of people who travel less frequently to meet others [33]. Recently, Xu at al. [34] quantified the temporal signature of geographical space in making friends by using cell phone records, suggesting that urban spaces and function distributions impact the shift of the spatial capability of connecting people.

Patterns of the physical co-presence or face-to-face encounters reflect the distributed urban vitality in public spaces and its publicness [22]. Two principle dimensions of face-to-face interactions, recognized in studies on physical interactions, are the distance-decay effect and spatial attraction [35]. This is in agreement with recent studies that use location-based “big data” to study mobility patterns [36–38] and to reveal various place attractions and competiveness by examining people’s traveling behaviors [39]. In these studies, the spatial configuration and functions (two essential elements operated in urban design and planning) are simultaneously found to be important in the manifestation of physical co-presence patterns. However, these studies were mainly conducted based on artificially-defined resolutions, e.g., grid cells or administrative areas, which are normally larger than the resolution in which urban forms are organized. Consequently, these approaches have rarely been implemented in urban design research and practice due to their low sensitivity to the change of urban forms and its geometric characteristics.

In the family of configurational studies, the effect of the geometry of urban grids on gathering people is focused. Specifically, in space syntax research, physical co-presence is a fundamental concept that interlinks urban geometric connectivity to its social effects by predicting the agglomeration of urban movement [40]. This has been verified by experiments with agent-based simulations [41] and by the good correlations between street network centralities and observed pedestrian flows [42]. Recently, Marcus [43] has re-conceptualized “spatial centrality” as “spatial capital” and emphasized its importance for understanding social performativity. Inspired by space syntax models, network-based geographical accessibility metrics for different sections of the population and job opportunities through spatial networks were employed to estimate co-presence potentials [44][45]. These studies have shed light on the interplay between physical co-presence potentials and geometric properties of the built public space; namely, the spatial conditions enabling the indivisibility among spatial agents, enriching the knowledge translation between the study of social interactions and urban design. These network-centrality-like measures, which are very sensitive to the modification of urban geometry, have been widely used by urban planners and designers to assess the social effectiveness of their spatial interventions [46]. Nevertheless, they are essentially static without the capacity for portraying the spatiotemporal evolution of the social interaction potentials. Consequently, it is still unclear how face-to-face interactions are facilitated geometrically by the built environment over time. As a result, quantifying physical co-presence potential explicitly by taking into account the influence of spatial geometries and its dynamic complexity, is a critical phase towards improving our understanding of encounters as environmental behaviors. In purely academic terms, this would be beneficial for morphological studies with emerging human activity data and space syntax research. In practical terms, this may be beneficial for appraising the dynamic functionality of space under specific spatial interventions, thereby enhancing the social effectiveness of the plans.

## Methods

### Research design

**Fig 1:**
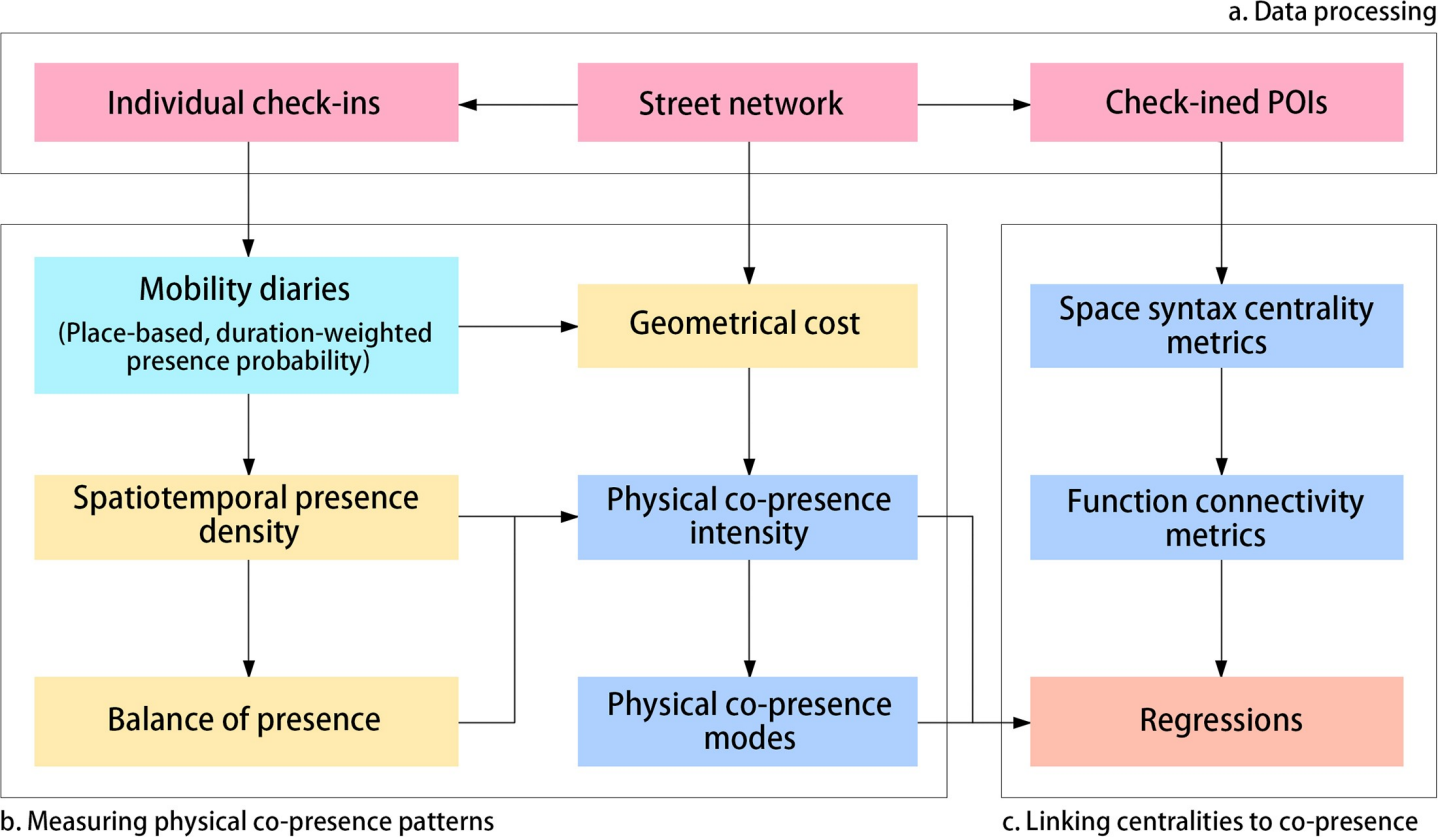
The research design.

The research flow of this study is charted in Fig 1, which can be divided into three phases: (a) data collection and pre-processing, (b) measuring face-to-face co-presence potential patterns, and (c) linking spatial centralities to co-presence. The first phase is designated for data collections and data processing to extract reliable information from the raw data gathered from an open data stream (open.weibo.com) provided by the largest Chinese social media service provider (Weibo). Specifically, the check-in records of anonymous social media users are filtered by eradicating invalid information, assigned spatially to the street network where the checked-in places are located, and aggregated successively to trips in order to produce objective descriptions of mobility patterns. In the second phase, we compute the physical co-presence intensity and its modes for various groups of social media users by packaging three elements, spatiotemporal presence density, balance of presence, and geometric cost, respectively representing the effects of density, diversity, and cognitive distance on the spatial interactions. The groups of social media users are distinguished into local and non-local groups by scrutinizing their mobility diaries and their physical co-presence probability turns to be the objects for the subsequent analyses. To explore the inherent meanings of the quantified results of the face-to-face co-presence over time as a series of new network centrality metrics, we test the explanatory power of these delivered measures on urban spatial structures through regressions where existing centralities of the spatial grid and land-use patterns are adopted as regressors. We use two different regression models: 1) a multivariate linear regression model to capture the ways in which urban space and land-use influence the structure of physical interactions on an hourly basis within a working day; and 2) a multinomial logistic regression to identify the contributions of the design of the built environment to the formation of the emerging physical co-presence modes in the streets.

### Measuring physical co-presence intensity through streets

#### Urban geometry in a People-Space-Time (PST) model

Physical co-presence, or a face-to-face encounter, is an urban phenomenon that can be conceptualized in multiple dimensions, each reflecting different types of “capital” related to people, space, and time. This study introduces an integrated model—the People-Space-Time model—in which the interactions between these types of capital can be comprehensively addressed. Each dimension of this model reflects the “distance” between two individuals in their environment, where the overall distance affects their likelihood of forming social ties [47]. The “people” dimension describes the “social difference” between two people. The “space” and “time” dimensions describe the physical separation of two people in space and time dimensions. Social difference fundamentally affects human interaction because it captures the internal variance between people. It is typically quantified using demographic features, such as age, social class, educational background, and income. It can also be defined by behavioral differences, for example, differences in traveling behavior. Spatial and temporal proximity are external conditions that affect people’s face-to-face interactions. Spatial proximity represents the spatial capital that people need to overcome in their trips to see one another. There are two types of spatial capital used in existing research: the “metric distance,” which refers to the geographical distance, reflecting the energy cost for people to travel to meet each other; and the “angular distance,” which captures the cognitive efforts that directly impact the mutual visibility between people in urban spaces. Essentially, the more two individual persons are metrically and geometrically proximal to one another, the more likely they will be to see one another. It has been argued that being metrically proximal is a precondition for a face-to-face meeting [33], but more importantly, people must be geometrically near enough at the same time [42]. Temporal proximity is self-explanatory: face-to-face meetings require people to be in the same place at the same time.

**Fig 2:**
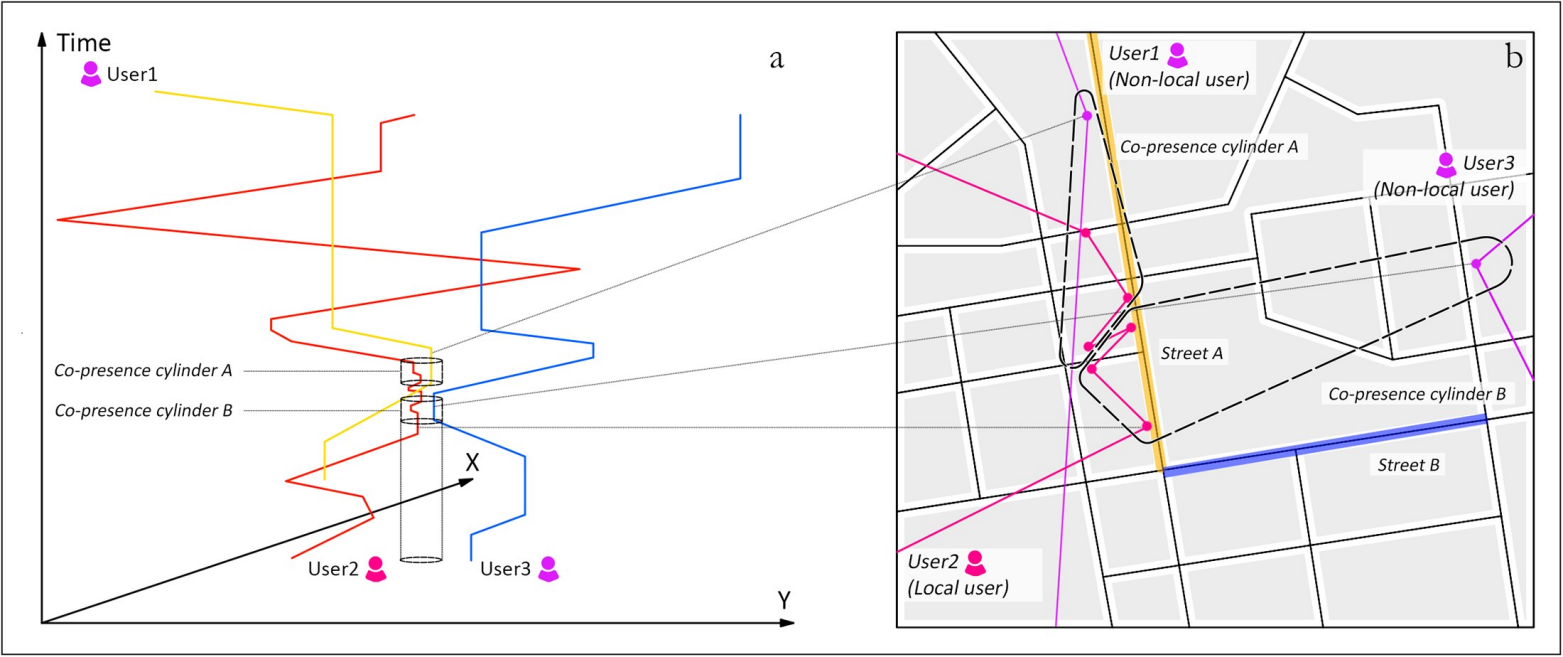
Interactions between people in the People-Space-Time (PST) model. (a) 3D spatiotemporal diagram plotting the trajectories of citizens as location coordinates (x, y) against time (z-axis). (b) Planar representation of the 3D spatiotemporal data in a, plotted onto a street-map.

In Fig 2a, the illustrative trajectories of three example users are mapped. Two co-presence cylinders are used to capture the co-location distributions between them occurring at different time periods. Cylinders A and B represent the areas for the occurrence of physical co-presence between subjects 1 and 2, and subjects 2 and 3, respectively. Within a typical spatiotemporal mapping system, based on Euclidean distance, these two cylinders might be very close. However, if a detailed planar representation of the spatiotemporal cube with the information of the spatial configuration is applied, as shown in Fig 2b, the geometric difference of co-presence cylinders emerges, which suggests that the cognitive cost further shifts the probability landscape for the actual face-to-face meeting through the built form by keeping the energy exposure to meet, reflected by the metrical proximity, constant. For instance, street A would be more likely to be the place where subjects 1 and 2 encounter one another, while street B should would be more likely for subjects 2 and 3 to encounter one another. However, since street B is further away from the actual subjects’ locations, street A would have a higher probability of two people meeting physically than street B. This example implies that the effects of the geometric distance should be considered beyond the metric distance when quantifying face-to-face interaction potential in the built environment, as the cognitive cost is key in the final stages of establishing face-to-face meetings. Meanwhile, addressing the geometry of urban built forms enables us to explicitly understand the functionality of urban design in conditioning social interactions in urban spaces.

### Measuring physical co-presence patterns

#### Defining subject groups using presence probability and duration

A central theme in measuring interaction potential is defining who it should be focused on, which allows the asking of questions with different aims at different levels. For example, the exposure index in segregation research addresses the geographical overlap of the daily activity space between selected populations defined based on the socioeconomic, ethnic, or demographic features of the residents. When selecting subject groups for this study, we focus on an important and fundamental factor in urban design, “the unstructured awareness of others,” which was argued to be a vital driver of urban vitality and is powerfully influenced by architectural forms [2]. Jacobs [22] has pointed out the necessity of creating diversity by clustering “strangers” to cultivate secondary relations between people—the familiarity generated by daily visual encounters rather than that produced by personal social networks—to maintain urban vitality. In the retail analysis, the transition density of the local and incoming flows was utilized to represent local and global place attractiveness [39]. Inspired by these ideas, for each location in the study area, we categorize subjects into two groups based on their movement around that location as characterized by social media check-in activity. For a given location *i*, local users are defined as subjects who walked randomly and frequently between locations in neighboring areas within a fixed time interval. Non-local users for location *i* are defined as subjects who visited the areas neighboring location *i* remotely from somewhere outside and left to go elsewhere after a very short stay. In short, for a local area during a certain period, the locals are mainly the parts of the internal flows within it, while the non-locals are manifested in the incoming flows to this area from and to somewhere else. For example, in Fig 2b, subject 2 is a local user who is frequently present in the streets near street A, whereas users 1 and 3 would be classified as non-local users to this location because they exhibit a much less frequent presence.

Based on these definitions of user groups according to their mobility variations, this study focuses on the relative perceived difference in terms of their travelling behaviors instead of the absolute inherent differences between people reflecting their social classes. In reality, few people can directly judge if the persons they encounter on the street are locals or non-locals. Instead, they could tell if they met someone somewhere. That is to say, people define others as locals or not according to their encounter frequency and duration, which are directly related to their mobility patterns in urban spaces. If someone is continuously present in a place, new visitors would feel familiar with him/her and then transform this awareness of familiarity in their cognition of the meeting place when they travel to that place at the same time.

**Fig 3:**
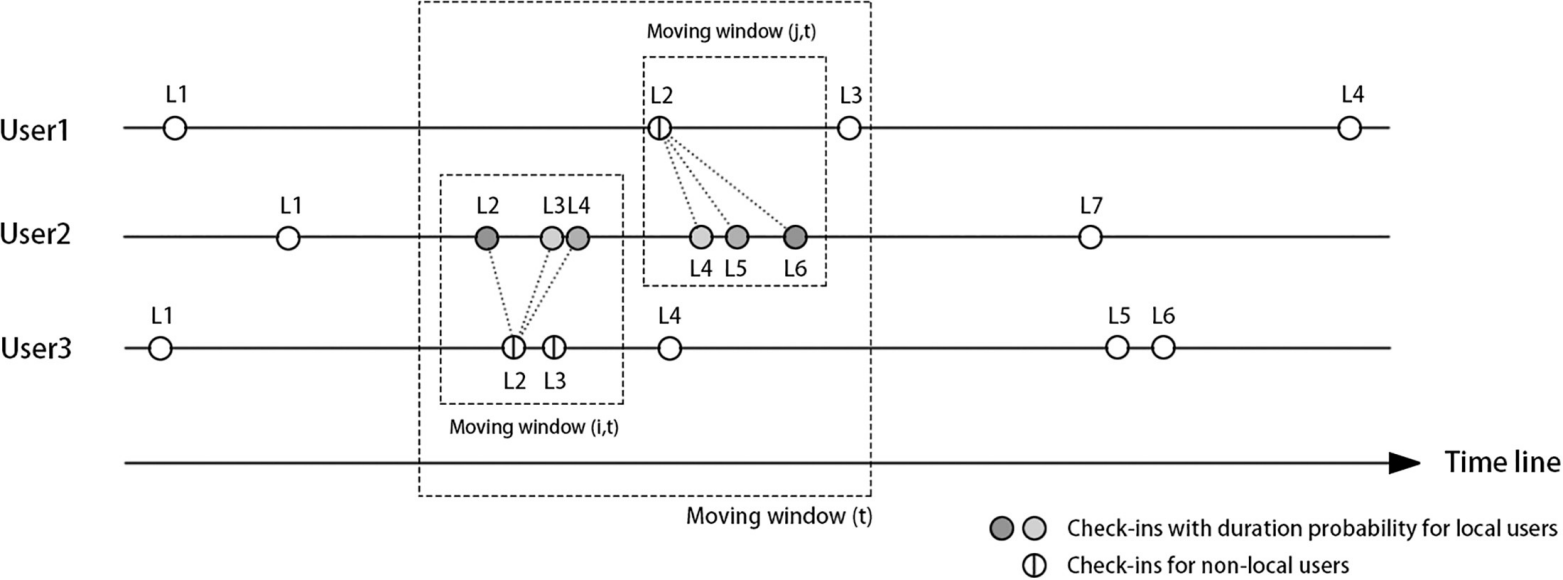
Definitions of space users based on their mobility diaries. (*L* refers to the location they checked in to; *t* denotes to the time period for defining a moving window which can be further specified according to the location *i* and *j*).

Fig 3 shows the procedure that we propose to define subject groups based on their trajectories as recorded in geo-referenced social media check-ins. A space-time moving window method is applied to classify subjects as “local” or “non-local” for a particular location. If a user appeared in one spatiotemporal window more than once and the total duration of stay was longer than a time threshold τ, he or she is defined as a “local” user. If users previously checked in at a window that is remote from the window of the location(s) of interest in question, and travelled to other windows afterwards in a very short period of time smaller than τ, they are labelled as “non-local” users for that location. This method produces a dichotomy between local and non-local valid space users. The interaction between the presence frequency and duration is used to detect these two user groups.

Given that the trajectories for a user *l* can be represented as a set of locations that were checked in to as a sequence $\left(L_{1}^{t_{1}},L_{2}^{t_{2}},\ldots ,L_{n}^{t_{n}},\ldots \right)$, the presence probability, $P_{l}^{\left(i,\Delta t\right)}$, that the user is at location *i* during a time interval Δ*t* can be expressed as a joint probability of two elements: the visit frequency, $P_{l}^{f(i,\Delta t)}$, and duration $P_{l}^{d(i,\Delta t)}$.

$$P_{l}^{\left(i,\Delta t\right)}=P_{l}^{f(i,\Delta t)}\times P_{l}^{d(i,\Delta t)}=P_{l}^{f(i,\Delta t)}\times \frac{\sum_{n}D_{l}^{\left(n,i,\Delta t\right)}}{D_{\Delta t}}$$ (1)

where $P_{l}^{f(i,t)}$ is a binary empirical probability, which equals 0 when user *l* visits the location *i* only once from somewhere else before Δ*t* ends, while it is 1 if they visit the location more than once. $P_{l}^{d(i,\Delta t)}$ is calculated as the cumulative duration of all visits to location *i*, expressed as a proportion of the total duration of the time period in question (*D*_Δ*t*_). Then, we can use a threshold μ to distinguish one group from the other. Here, we define the users with presence probabilities greater than or equal to 0.5 as local users (μ = 0.5), and those with presence probabilities of less than 0.5 as non-local users. Given this, a non-local user for a place during a time period either visit it only once before leaving or visit it several times in a very short period of time (τ = 0.5Δ*t*). It is assumed that the duration of the *n*^th^ stay ($D_{l}^{\left(n,i,\Delta t\right)}$) at location *i* during time period Δ*t* was equal to half the time period between its antecedent and subsequent check-ins ($D_{l}^{\left(n,i,\Delta t\right)}=\frac{t_{l}^{\left(i,n+1\right)}-t_{l}^{\left(i,n-1\right)}}{2}$). The location *i* here can be identified according to the chosen resolution. In this study, it was defined as the neighboring zone centered at the street segment *i* at a network radius *r*.

#### Physical co-presence density

After assigning subjects to groups, we quantify the agglomeration of human flows in each user group at each location by measuring the cumulative amount of trips, weighted according to their duration. The index of “*physical co-presence density”* (${Den}_{i}^{\Delta t}$) can be formally expressed as a sum of the “*physical presence density*” for each group (${Den}_{i}^{\left(k,\Delta t\right)}$) by the equation below.

$${Den}_{i}^{\Delta t}=\sum_{k}{Den}_{i}^{\left(k,\Delta t\right)}=\sum_{k}\sum_{j}\sum_{l}\sum_{n}D_{l}^{\left(k,n,j,\Delta t\right)},{\;\{d}_{ij}\leq r\}$$ (2)

Here,${Den}_{i}^{\Delta t}$ is the weighted number of trips happening in the neighboring area of street segment *i* at a fixed radius *r*. ${Den}_{i}^{\left(k,r,\Delta t\right)}$ is the number of trips for the user group *k*. $D_{l}^{\left(k,n,j,\Delta t\right)}$ refers to the duration of stay by subject *l* in group *k*, checked-in at location *j* during the time period Δ*t*, and *d*_*ij*_ denotes the network distance along the street network. This index measures the levels of the co-location between various groups based on the metric distance, which is a precondition for their face-to-face encounters.

#### Balance of physical co-presence

Here we define another index, *B*, which measures the balance between local and non-local groups in terms of their physical presence densities. Normalized information entropy is applied to quantify the degree of balance. Assuming there are *K* (*k* = 1, 2, 3, …, *K*) groups of users in question, we calculate the temporal presence probability ($p_{i}^{\left(k,\Delta t\right)}$) for each group by subdividing its standardized presence density (${NaDen}_{i}^{\left(k,\Delta t\right)}={Den}_{i}^{\left(k,\Delta t\right)}/\sum_{i}{Den}_{i}^{\left(k,\Delta t\right)}$) by the total normalized presence density of all groups. In this study, only two social groups were classified: *k*_1_ = local and *k*_2_ = non-local.

$$B_{i}^{\Delta t}=\frac{\sum_{k}p_{i}^{\left(k,\Delta t\right)}\times \text{log}\;p_{i}^{\left(k,\Delta t\right)}}{-\text{log}\;K},{\{d}_{ij}\leq r\}$$ (3)

$$p_{i}^{\left(k,\Delta t\right)}=\frac{{NaDen}_{i}^{\left(k,\Delta t\right)}}{\sum_{k}Na{Den}_{i}^{\left(k,\Delta t\right)}}$$ (4)

#### Cognitive cost

As stated, cognitive cost projected in the built environment is an essential factor in the finalization of face-to-face encounters beyond the co-location phenomenon [2]. We use the mean angular change along the shortest path (${MD}_{i}^{\Delta t}$) as a measure of the cognitive effort required to connect two individuals in a physical space. This can be formally defined by,

$${MD}_{i}^{\Delta t}=\frac{\sum_{j}{ad}_{ij}^{\Delta t}}{J},\;{\{d}_{ij}\leq r\}$$ (5)

where ${ad}_{ij}^{\Delta t}$ represents the cumulative angular distance between street segment *i* and the reachable check-in location *j* along the shortest path connecting them, and *J* is the total number of check-ins assessable from street *i* at a radius *r*. We can also calculate this for specific groups of subjects by simply converting Eq 5, above, to a more disaggregate form:

$${MD}_{i}^{\left(k,\Delta t\right)}=\frac{\sum_{j}{ad}_{ij}^{\left(k,\Delta t\right)}}{J},\;{\{d}_{ij}\leq r\}$$ (6)

#### Physical co-presence intensity through streets

The physical co-presence intensity index ($I_{i}^{\Delta t}$) measures the extent to which various groups of people cluster physically and geometrically in a local area around street *i* within radius *r* within a given time interval Δ*t*. It combines the three indices introduced above into one integrated measure. Given this, we conceptualize the physical co-presence potential as the interplay among the presence density, cognitive distance and their balance with the people, and space and time constraints.

$$I_{i}^{\Delta t}=\frac{{{Den}_{i}^{\Delta t}}^{B_{i}^{\Delta t}}}{{MD}_{i}^{\Delta t}},{\{d}_{ij}\leq r\}$$ (7)

In this equation, we utilize the balance index, $B_{i}^{\Delta t}$, as a punishing power which will transfer the co-location potential to a more social format. The presence density will be decreased with the drop of the evenness of its composition. This equation can be thought of as being similar to a typical model of gravitation, in which the potential of spatial interactions is positively related to the magnitude of attractiveness but negatively linked to spatial proximity. The difference between our model and a gravity model is that we address the diversity effect and simultaneously consider geographical and geometric distance. Eq 7 can be disaggregated by social groups to produce a physical presence intensity for each group by removing the balance index, as follows:

$$I_{i}^{\left(k,\Delta t\right)}=\frac{{Den}_{i}^{\left(k,\Delta t\right)}}{{MD}_{i}^{\left(k,\Delta t\right)}},{\{d}_{ij}\leq r\}$$ (8)

In the equation above, the resulting index estimates various types of the within-group co-presence potentials. If *k*_1_ = local, the output shows the interaction potential between the locals, a description of the space’s capacity for sustaining random local visits in the streets; if *k*_1_ = non-local, the resulting index illustrates the global attractiveness of the place *i* featured by the interaction potential between non-local users. As such, the index of the physical co-presence intensity for various groups as shown in Eq 7 can be considered as a new form of place attractiveness that combines the place capacity of sustaining random local visits, and attracting people from other places.

We close this section by summarizing the procedure of computing the indices delivered, step by step (S1 Appendix). The start points are the street network graph data (G) and trajectory data (T). 1. Select a segment in G; 2. Use a space-time filter (r, Δt) to search for all check-in points within that space-time frame; 3. Classify the users (L) with the threshold (μ) based on check-in times and duration; 4. Calculate the indices proposed for the whole sample and each group according to the equations shown above; 5. If all the segments are processed, stop. Otherwise go back to 2.

#### Detecting the mode(s) of physical co-presence

Although the proposed physical co-presence potential index is a reasonable summary of the interplay between various forms of social interaction potentials, under different social, spatial, and temporal conditions, it may simplify some detailed differences in the co-presence patterns that are hidden behind the produced scored landscape. Therefore, to capture the spatiotemporal co-presence modes across streets, this research uses a clustering analysis to group streets according to the observed difference in the presence density of and cognitive cost between the defined social groups of people. This analysis applies the k-means clustering analysis with the principal components derived from the raw data to determine the proper membership for each street. Pseudo *F*-statistics are adopted to judge the performance of the clustering and select the most appropriate number of clusters.

#### Parameters

In this study, we select street segments as the basic units for analysis. A street segment is the public space between two street intersections. Street segments are real spaces where face-to-face co-presence occurs with metric and geometric distance, and they are vector-based, meaning that they are not susceptible to the modifiable areal unit problem that would otherwise impact the robustness of the analysis. The other two basic parameters used in the analysis are the radius *r*, for defining the local area of the segment *i*, and the time interval Δ*t* for identifying the time resolution. We use a 750 m walking distance as the radius, *r*, defining the local area around segment *i*, based on an assumption that the average walking speed is approximately 5 km/h and the average walking time is 9 min [48][49]. We use a time interval, Δ*t*, of one hour Additionally, one hour is used as the interval because it was found that one hour is an optimized time scale for the proposed analysis; making the time scale smaller would risk compromising the reliability of the data since the average sample size would be also be smaller. Nevertheless, such a fine-scale co-presence pattern may generate bias with a large amount of variability but less regularity, thereby constraining the production of reasonable patterns [50]. Therefore, a time interval of one hour was chosen to provide a good balance between temporal singularity and regularity.

### Linking network centralities to physical co -presence potentials

The proposed indices that were calculated and mapped in the street network show a series of centrality-like descriptions of urban spatial structures characterized by the physical co-presence potentials. They can be compared with the space syntax centrality measures that were proposed to investigate the performance of the introduced metrics as the dynamic space syntax metrics, and reveal the relationship between the spatiotemporal interaction potentials and the arrangement of urban forms and functions.

#### Indexing the centralities of the spatial configuration

The spatial configuration contains two interdependent sub-systems: the spatial network and land-use patterns. By converting these two systems into graph-based representations, we compute the graph centralities of these two systems separately, including space syntax centrality and urban function connectivity measures. The former measures the shallowness between two spaces, while the latter covers critical aspects of relatedness between urban functions along spatial grids.

#### Regression analyses

To explore the interrelationships between static network centrality measures and the dynamically changing network potentials of the co-presence intensity and the related community structure, a multivariate linear regression and a multinomial logistic regression model are employed (Table 1). This study employs the stepwise technology in these two regression models to select the important factors determining the observed variation of the dependent variables by filtering out the factors with less contribution to the model goodness-of-fit. This method can efficiently control the risk of over-fitting and produce an essential variable structure. Before the stepwise variable filter method is applied, the variables maintaining a higher risk of multicollinearity are detected and removed. The principle is defined by setting the threshold of the variance inflation factor (VIF) for each variable. In this study, the variables with VIF values larger than 10 are removed from the models.

**Table 1:** Centrality measures of spatial configuration.

	Abbreviation	Definition
*Space syntax centrality measures*		
Integration	INT %Radius%	The angular closeness of street network at a radius
Choice	CHO %Radius%	The angular betweeness of street network at a radius
*Urban function centrality measures*		
Density	DEN %Radius%	The accessible function density of POIs through the street network at a radius weighted by place-based social media check-ins
Diversity	DIV %Radius%	The accessible function diversity of POIs through the street network at a radius weighted by place-based social media check-ins
Distance	DIS %Radius%	The mean angular step depth to the reachable POIs through the street network at a radius

## Materials

### Study area

**Fig 4:**
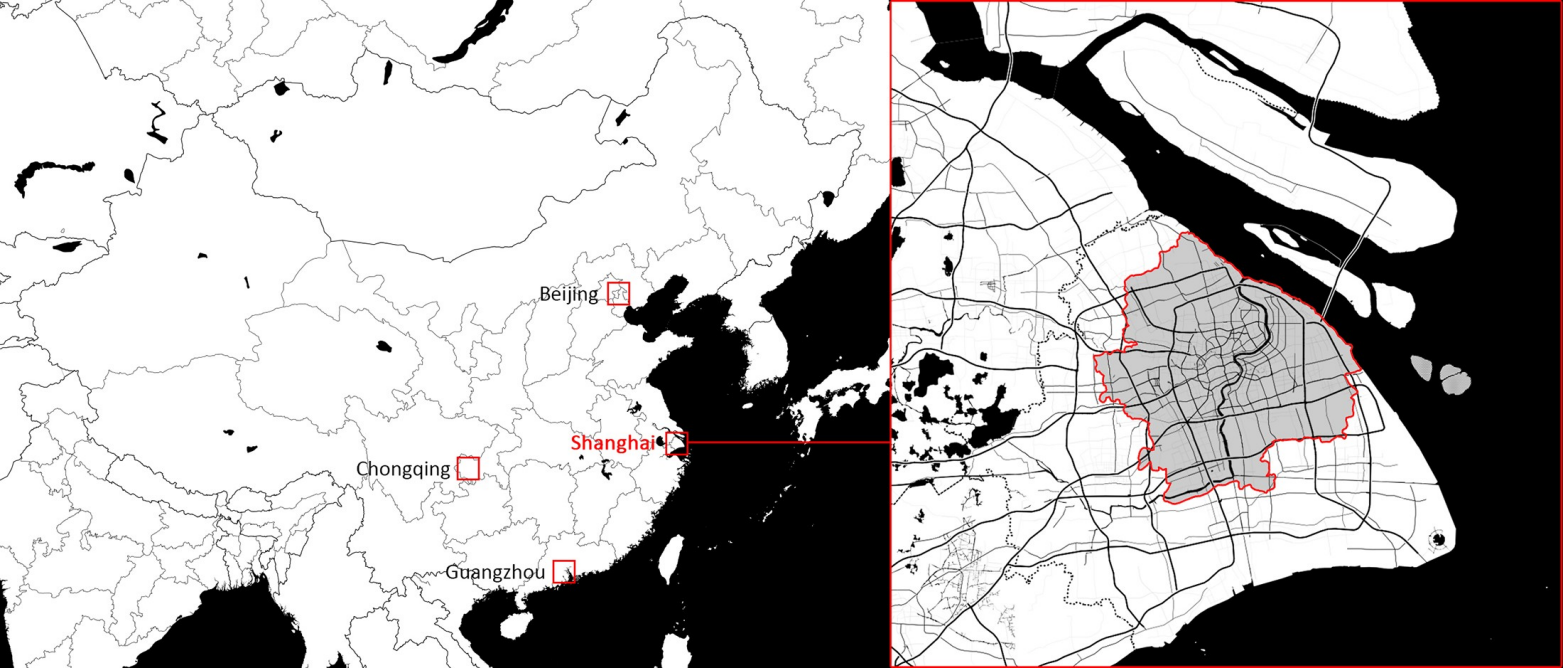
The study area.

Central Shanghai, the spatially continuous areas with population density larger than 3,000 per square kilometers, is selected as the case for the empirical investigation in this research (Fig 4). In accordance with the pattern of recent rapid economic growth in China, Shanghai has been growing dramatically since the 1980s. The urbanization process in Shanghai can be described as “Chinese modernization in miniature.” The spatial expansion and the shift of the spatio-social structures provide an ideal case to examine the interactive relationship between the spatial form and temporal social processes. Meanwhile, as one of the most developed cities in China, Shanghai maintains the largest group of social media users due to its large size of population and a high rate of social media penetration, which enables the presupposition of using the social media dataset to precast people’s movements within the city.

### Trajectories in social media check-in data

**Fig 5:**
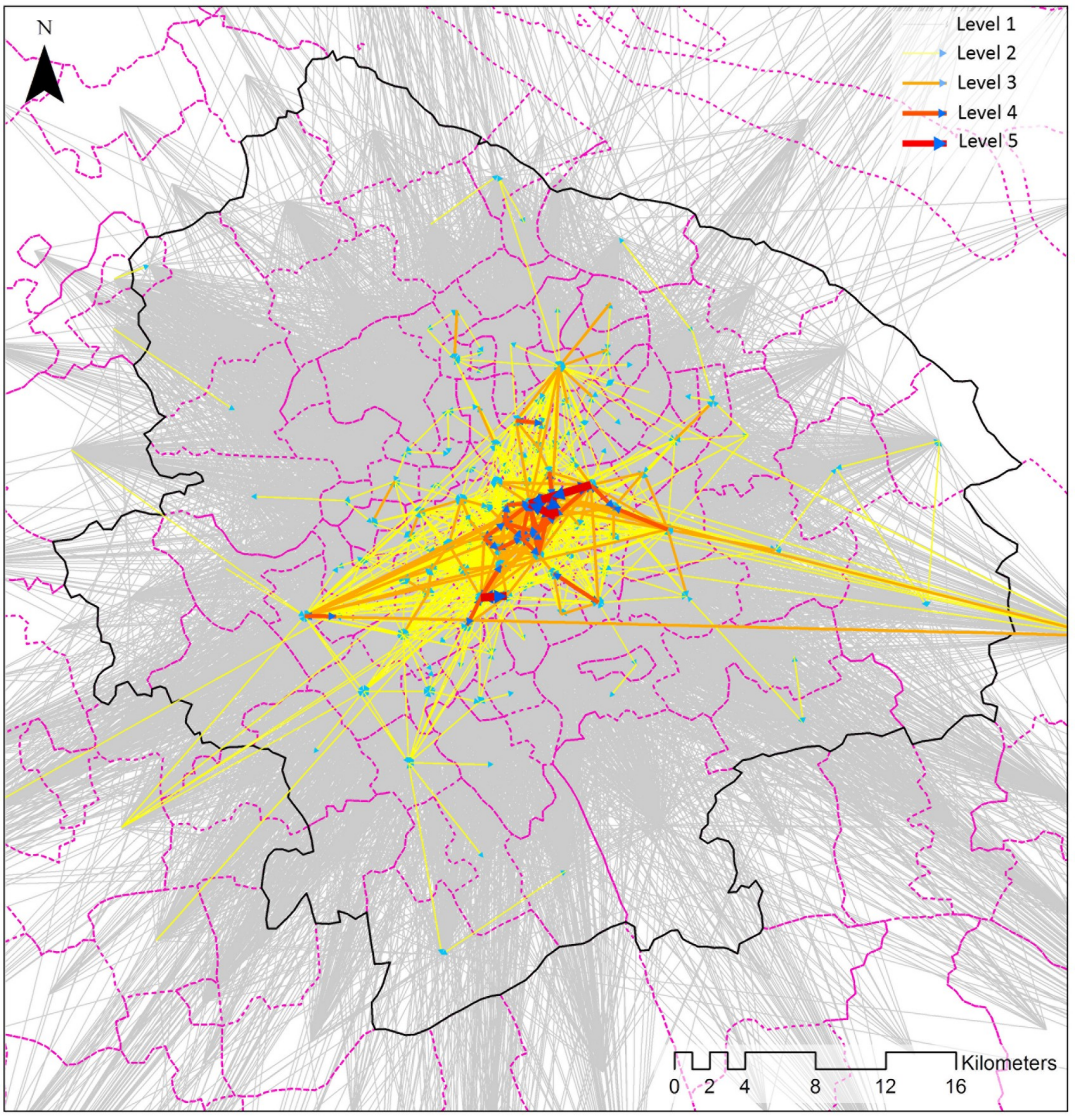
Aggregated trips between census units in Central Shanghai.

The intra-city trajectory patterns of social media users are extracted from individual check-in records collected on workdays for a quarter-long period from March to May 2016. The raw dataset included 2,868,972 records from 73,427 users across 48,234 venues within the boundary of the study area. Even though social media check-in records are a type of fine-scale location data, they cannot be directly used to estimate real mobility distributions because of the existence of fake check-in records. We therefore apply a set of rule-based pre-processing steps to produce a cleaner dataset of the movements of individual social media users on a typical workday. These steps were taken as follows: 1) removing invalid check-in records in which the actual locations of the smartphone users do not match the locations of the venues to which they want to check in; 2) eradicating the check-ins that are more than 20 meters away from their nearest street segment; 3) removing the users who have only checked in once; 4) producing spatiotemporal trips of a person based on his/her consecutive check-ins; 5) eliminating anomalous trips with unexpected travel speed or duration (the thresholds of speed and duration are 400 km/hour and 12 hours, respectively); 6) merging extracted trips into a typical workday (the initial check-in trajectories for a user on different days are segmented as substantive groups with unique IDs); and 7) eradicating the trips that do not move towards the locations in the study area. Our final dataset for further analysis consisted of 584,746 trips towards destinations in Central Shanghai. The aggregated results between the census units are shown in Fig 5, in which the directional polycentric structure is illustrated.

### Gate counts

**Fig 6:**
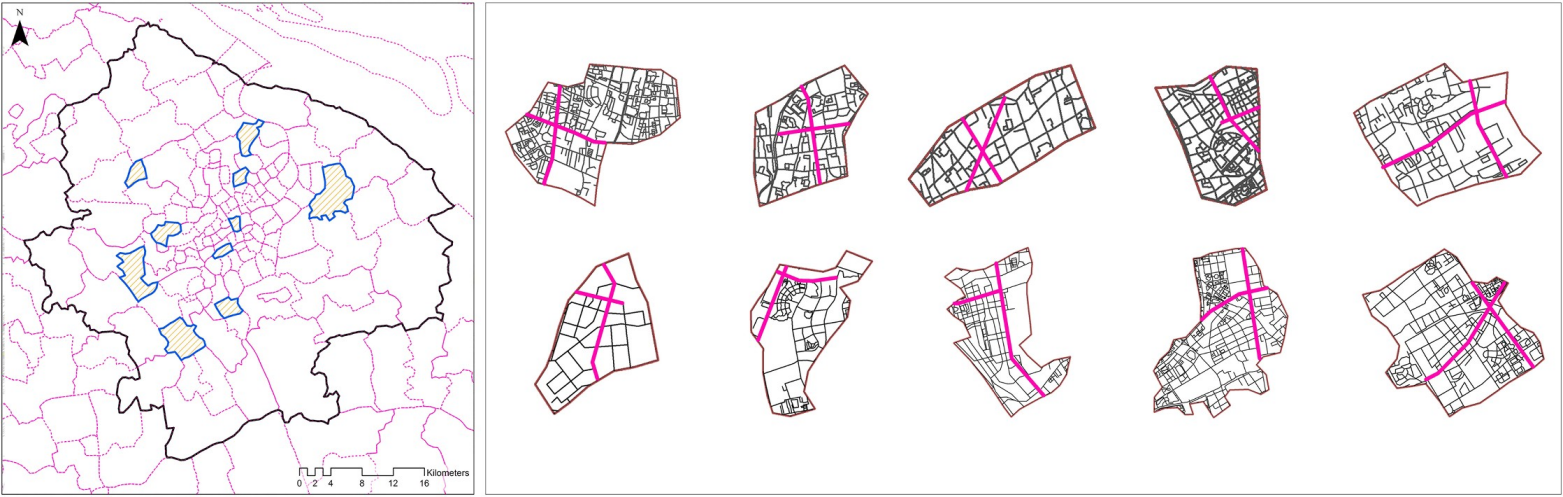
Main streets (in fuchsia) in ten census areas randomly selected from Central Shanghai.

Gate count data were collected on several workdays in November 2016 from the main streets in ten randomly-selected census areas (Fig 6). At each gate for an individual street segment, the aggregated flows were counted over three hour-long periods: from 9:00 to 10:00, 14:00 to 15:00, and 21:00 to 22:00. The data were collected to test the reliability of social media check-in data for describing urban movement, and to validate the effectiveness of the proposed method to quantify physical co-presence potentials.

## Results

### Preliminary validation

Before the introduction of the empirical results, one primary task is to validate the reliability of the extracted trajectories and the relationship between the computed patterns of the spatiotemporal co-presence potential of the online population and the actual agglomeration of people on the streets. This aim is achieved in the present study by conducting two comparisons. The first concerns the scaling nature of the mobility data in social media check-in records, while the second concerns the goodness of the correlation between the calculated co-presence intensity and the surveyed gate counts. In this regard, this study verifies the effectiveness and applicability of the proposed methods with social media data.

#### The scaling nature

**Fig 7:**
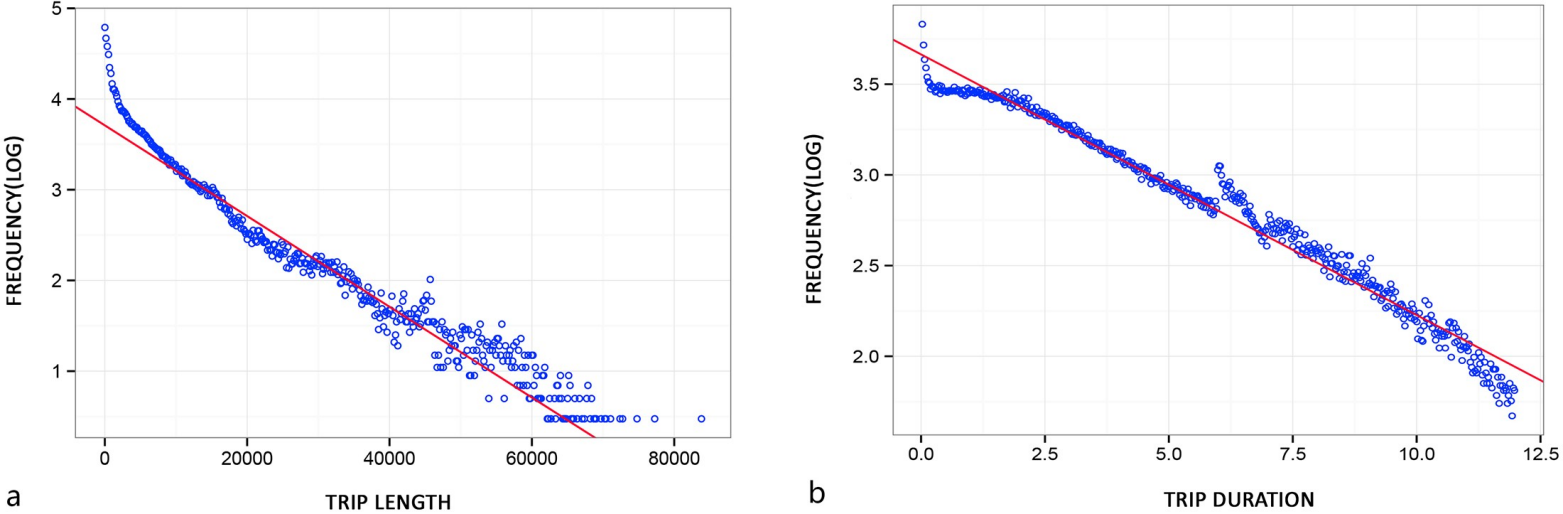
Distributions of trip length and duration. (a) Semi-log scatter plot of the distribution of trip length in the dataset. Red line shows best-fit exponential function. (b) Semi-log scatter plot of the distribution of trip duration in the dataset. Red line shows best-fit exponential function.

It has been widely discussed that scaling phenomena are common in mobility patterns. The scaling property of a distribution can be specified and modelled in several ways. For instance, data can be fitted by a power law function (*f*(*x*)~*kx*^*-β*^) or an exponential function (*f*(*x*)~*e*^*-αx*^). We tested fitting with both types of function, and found that individual movement records in social media check-in data can be best fitted by exponential functions. The trip length distribution is well-fitted (*R*^*2*^ = 0.952) by a function with exponent *α* = 0.121 (Fig 7a), and trip duration distribution is well-fitted (*R*^*2*^ = 0.977) by a function with exponent *α* = 0.144 (Fig 7b). These results are in accordance with the findings of previous studies [11][38][51]. This is evidence that the extracted trips in this study maintain the inherent scaling structures of human movement data, supporting the feasibility of using social media check-in records for such studies.

#### Correlation with observation

**Fig 8:**
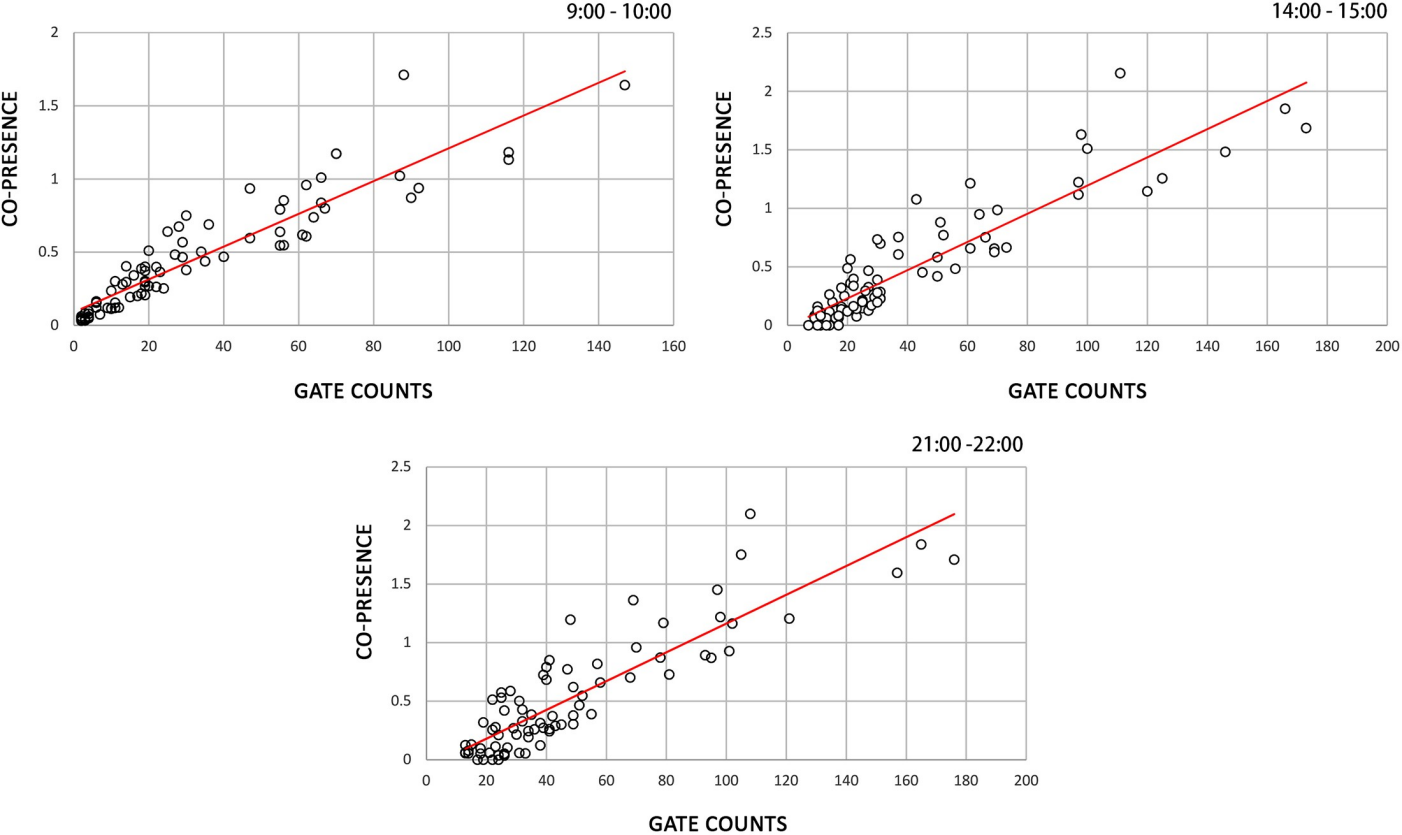
Scatter plots of gate counts against physical co-presence intensity. (a) Time period, 9:00–10:00; R2 of linear fit = 0.852. (b) Time period, 14:00–15:00; R2 = 0.821. (c) Time period, 21:00–22:00; R2 = 0.807.

Physical co-presence distributions are rooted in urban mobility distributions. Despite the fact that co-presence patterns are more complex than the flow size distributions, the urban flow size is still the primary factor determining the underlying probability of people’s physical interactions. Spatially varying co-presence patterns should therefore be reasonable estimations of pedestrian flows. Consequently, the accuracy of the produced results is evaluated preliminarily by the examination of their correlation with the survey data. Fig 8 illustrates the scatter plots in which the gate count is understood to be a function of the co-presence variable over three time periods. The results indicate that the spatiotemporal co-presence patterns generated by the proposed method are highly correlated with the survey data and this trend is stable across time, with R-square values larger than 80%. These findings show that the dynamic co-presence patterns can not only capture the spatial discrepancy of urban flows, but also portray their temporal disparity.

Even though the scaling nature of human dynamics and a good correlation between the proposed indicators and the observation can be detected, there might be potential bias of connecting local and non-local individuals by social media check-in records. Social media data may be a biased representation of the entire population and the check-in habits of social media users might be different from that of their actual daily dynamics [5]. According to the proposed definitions of the local and non-local in this paper, the frequent social media users might be more likely to be defined as the local people. To solve this problem, a cross-validation based on multiple-data could be done if possible, which could contribute to calibrating a reliable threshold μ of presence probability and a proper buffer distance *d*_*ij*_ to well distinguish these two groups of space users.

### Physical co-presence intensity patterns

**Fig 9:**
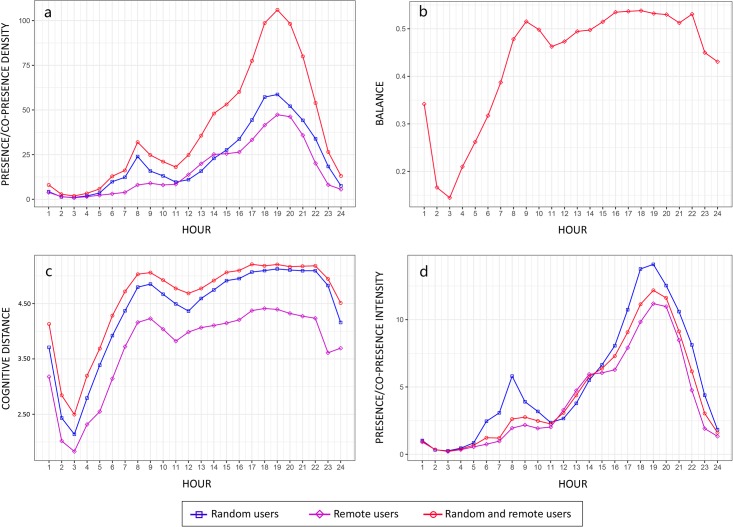
Change of the average presence/co-presence measures across time in Central Shanghai. ((a) Presence density; (b) Co-presence balance; (c) Mean cognitive distance (angular step depth); and (d) Presence/co-presence intensity).

Fig 9 describes the temporal changes of the introduced measures regarding various aspects of physical presence/co-presence potential. The pattern of presence density demonstrates that the check-in behaviors of social media users tend to be more frequent in the evening (Fig 9a). Moreover, the average degree of the clustering of local users in the streets is higher than that of the agglomeration of non-local users during most time periods with the expectation that the latter is slightly stronger than the former from 12 am to 2 pm. The temporal shift of the balance degree is captured in Fig 9b, in which the interaction between the presence of local and non-local users is lowest at 3 am and moves to over 0.4 after 8 am. The lower values observed before 8 am indicate the spatial differentiation between local and non-local people flows because many trips occurring during this period are towards residential communities where few people will be active at typical sleeping times. The balance index then decreases to 0.45 at 11 am and increases back to 0.5 after 2 pm. This may result from the reallocation of destinations during lunch time. Similar to the trend observed in the change of the balance index across time, the value of the cognitive distance for the presence of both non-local and local users reaches the lowest point and moves to the peak, but a similar dip is also observed around lunch time (Fig 9c). This trend demonstrates that the presence of people is more geometrically concentrated in some places, but is more dispersed from a city-wide perspective during the periods when the presence density and balance degrees are temporally lower.

It can also be observed that the average cognitive distance for all users is always higher than the mean angular distance between people in the same group. Furthermore, the mean angular distance for local users encountering one another in the street is higher than that of non-local users, which suggests that destinations for non-local users are more configurationally closed, whereas the journeys’ ends for local walkers are geometrically scatted but are metrically concentrated. The maps of presence/co-presence intensity are illustrated in Fig 9d. The gap between the presence patterns of local and non-local users in terms of presence density is shortened when the cognitive distance is taken into account. The co-presence intensity index exhibits a relatively smooth change and is located in the interval between the two presence intensities of individual groups. In short, the significant fluctuation of the presence and co-presence intensity patterns reveals the temporal complexity of co-presence patterns which is difficult to capture in aggregated descriptions of urban flows.

**Fig 10:**
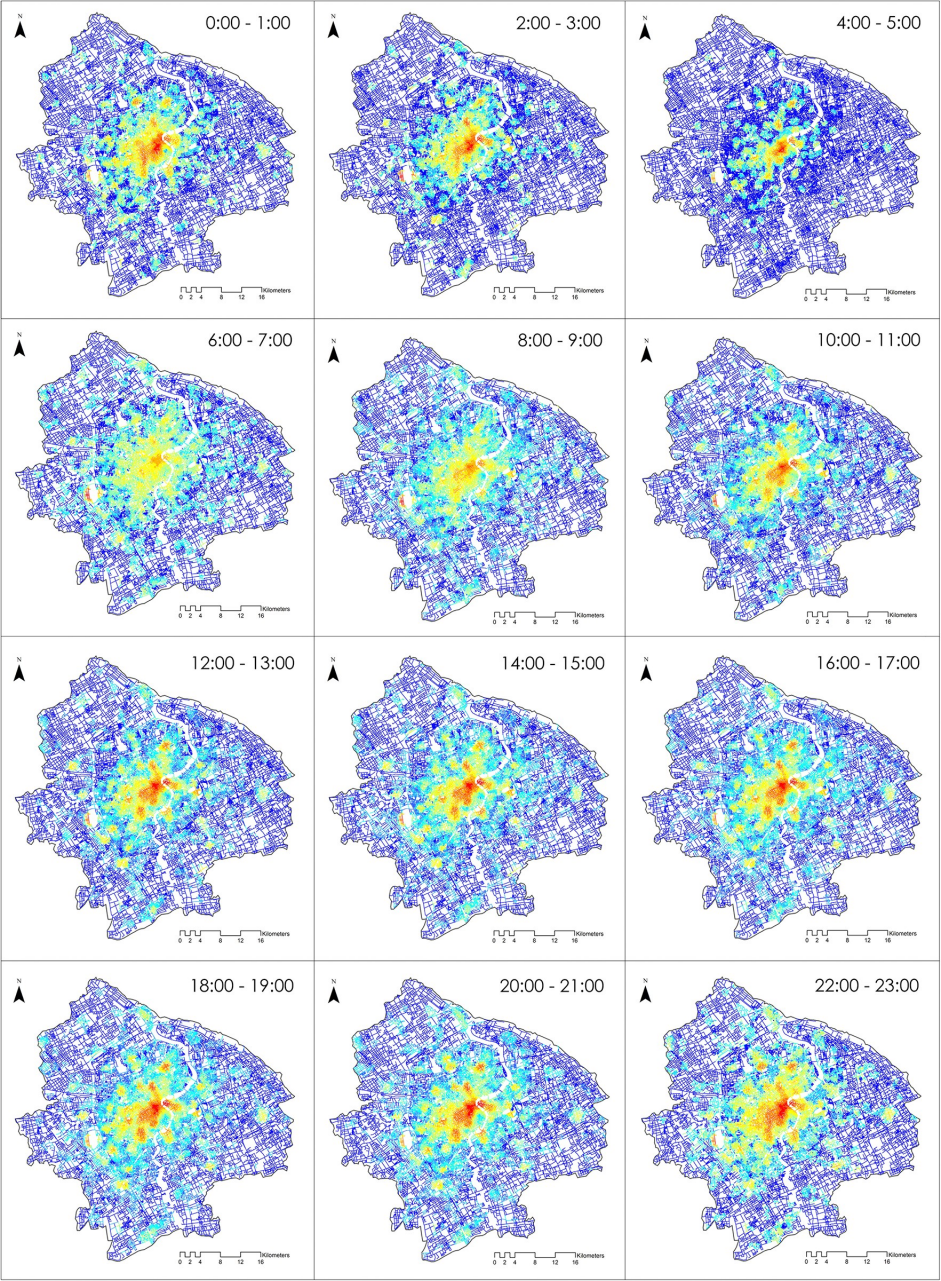
Physical co-presence intensity maps in Central Shanghai.

The spatiotemporal change of the co-presence intensity is mapped in Fig 10 using the same symbolizing method. The overall urban polycentric structure can be discovered across time based on visual judgment, although the shape of the co-presence cores changes dynamically. This suggests that the face-to-face co-presence pattern has its roots in the urban structure. In the early morning, the co-presence pattern becomes compact around the city centers, particularly from 4 am to 5 am. When commuting time approaches, the co-presence intensity becomes more spatially homogeneous since people are travelling to workplaces that are distributed in a more scattered manner. The global city center regains its dominance after 9 am, and this trend remains significant during the rest period. Notably, some locations are also highlighted during an all-day period. Hongqiao transport hub, for instance, maintains a high degree of physical co-presence values at all times. This is evidence that modern mix-used complexes, such as transport centers, shopping malls, etc., and the streets connected to them are emerging places for human interaction.

**Fig 11:**
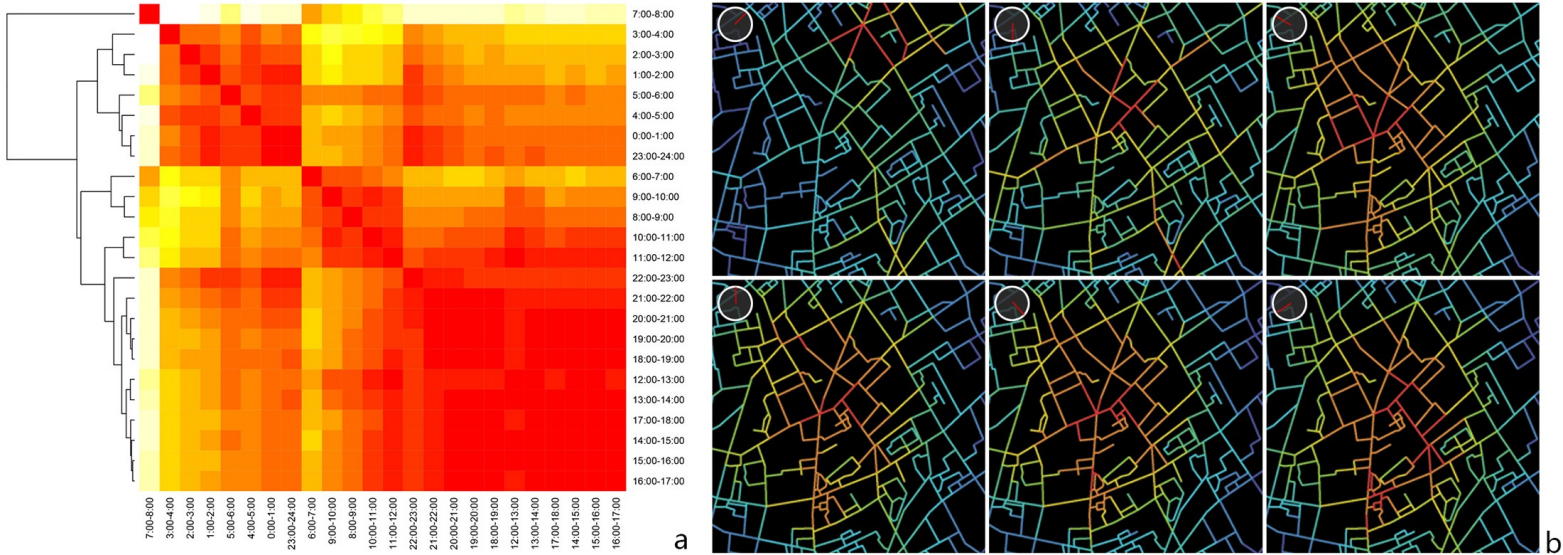
Correlation heatmap of the physical co-presence intensity indices (a) and physical co-presence intensity maps of a selected area (b).

The correlative relationships among the physical co-presence intensity indices across time indicate the spatiotemporal association of the physical co-presence intensity scores (Fig 11a). Apparently, the distribution of the co-presence potential in the morning peak between 7 am and 8 am is different from that during other periods. The potentials in the early morning are significantly temporally-dependent. And the score for the late morning is another typical time-period when the structures of the co-presence potentials are relatively stable. After the lunch-time, another trend emerges and continues until the mid-night. This global tendency, however, is not always held locally. Fig 11b represents a selected area in Shanghai where the centrality of physical interaction potentials shifts fast from the high streets to the back through the public space system. This also suggest that the method introduced can be adopted as a tool to track the temporally changing contribution that urban configuration makes to facilitate interactions. Once the spatial layout is redesigned, the co-presence potential patterns are transformed accordingly.

**Fig 12:**
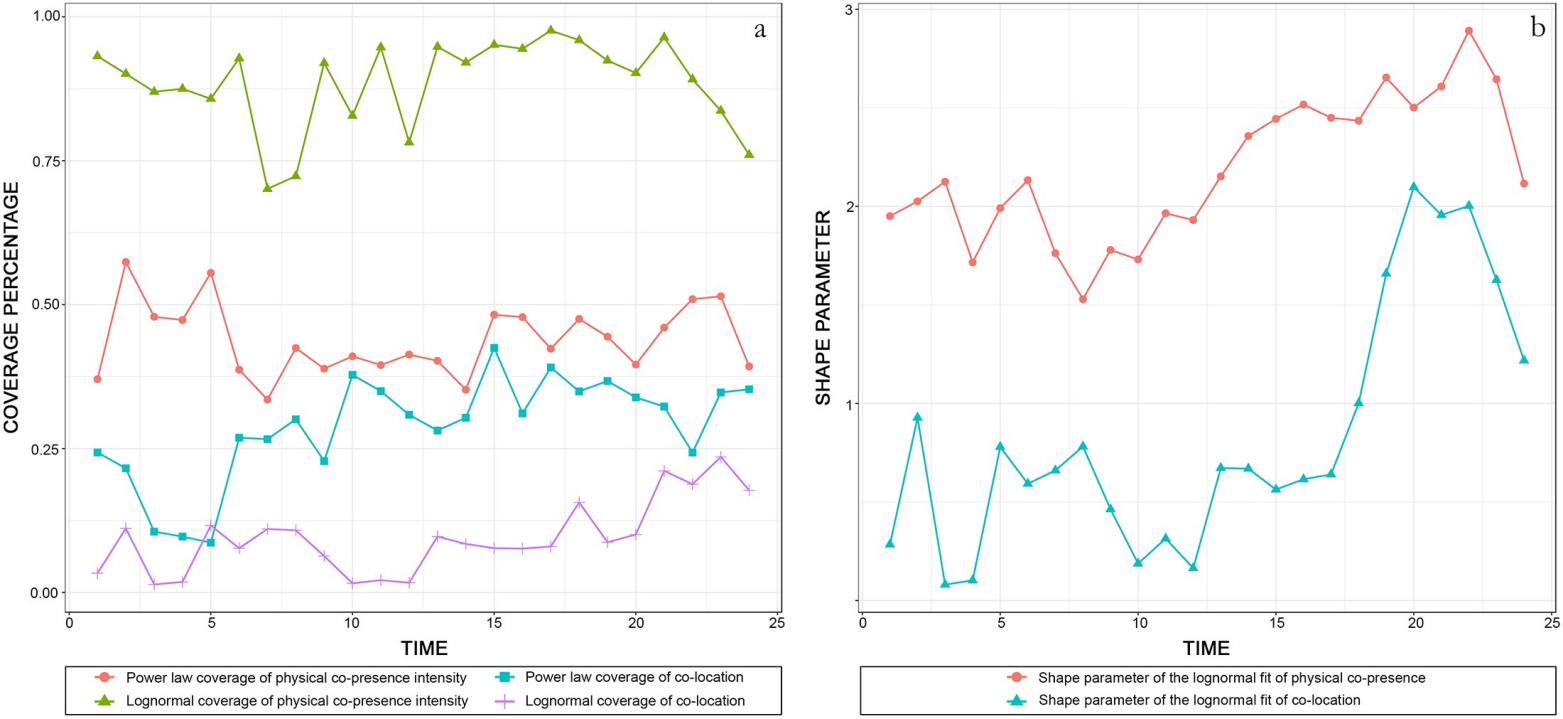
Changing performance of different fitting models of physical co-presence intensity patterns and co-location distributions across time. ((a) change of the coverage percentage for power-law and lognormal fits over time; (b) change of shape parameter of lognormal fits over time).

It is noticed that the proposed measure of the physical co-presence intensity does not follow any specific function globally, but it follows some for a continuous subset of the whole sample with the scores larger than a detected critical value. Fig 12 illustrates the changing performance of different fitting models of the introduced indices across day-time, compared with the fitting distributions of the co-location patterns captured by the presence densities. It is observed that the physical co-presence intensity index over periods can be described by a lognormal distribution with larger shape parameters and wider fitting coverage shares (the percentage that the selected sample occupied) than the co-location potentials measured by the geometric agglomeration of place users using the same radius to define the buffer zones. This indicates that the face-to-face co-presence patterns follow heavy tail distributions that are more left-skewed than the co-location patterns. It is evidence that face-to-face encounters, compared with co-locations, concentrate in fewer streets, which is appropriately addressed by the delivered measures where geometric cost is considered.

### Physical co-presence modes

**Fig 13:**
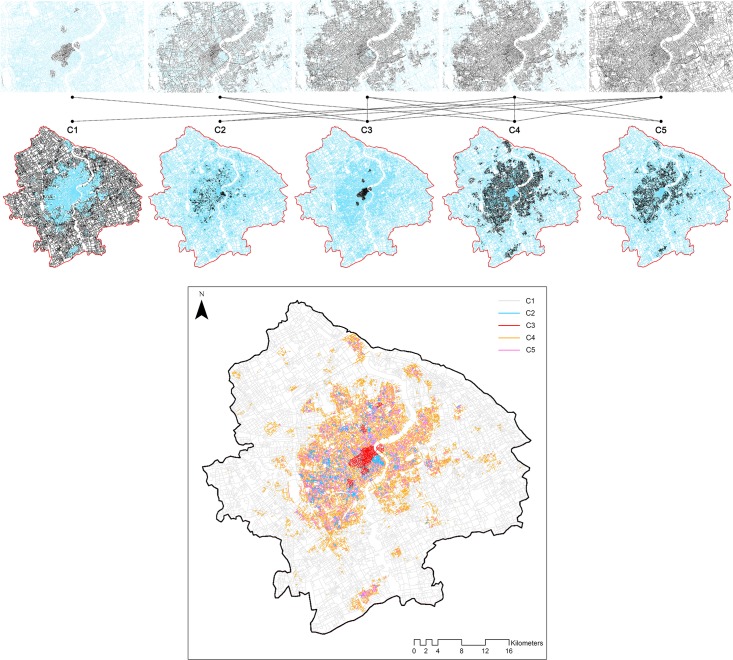
Detected clusters based on the modes of spatiotemporally changing physical co-presence indices in Central Shanghai.

**Fig 14:**
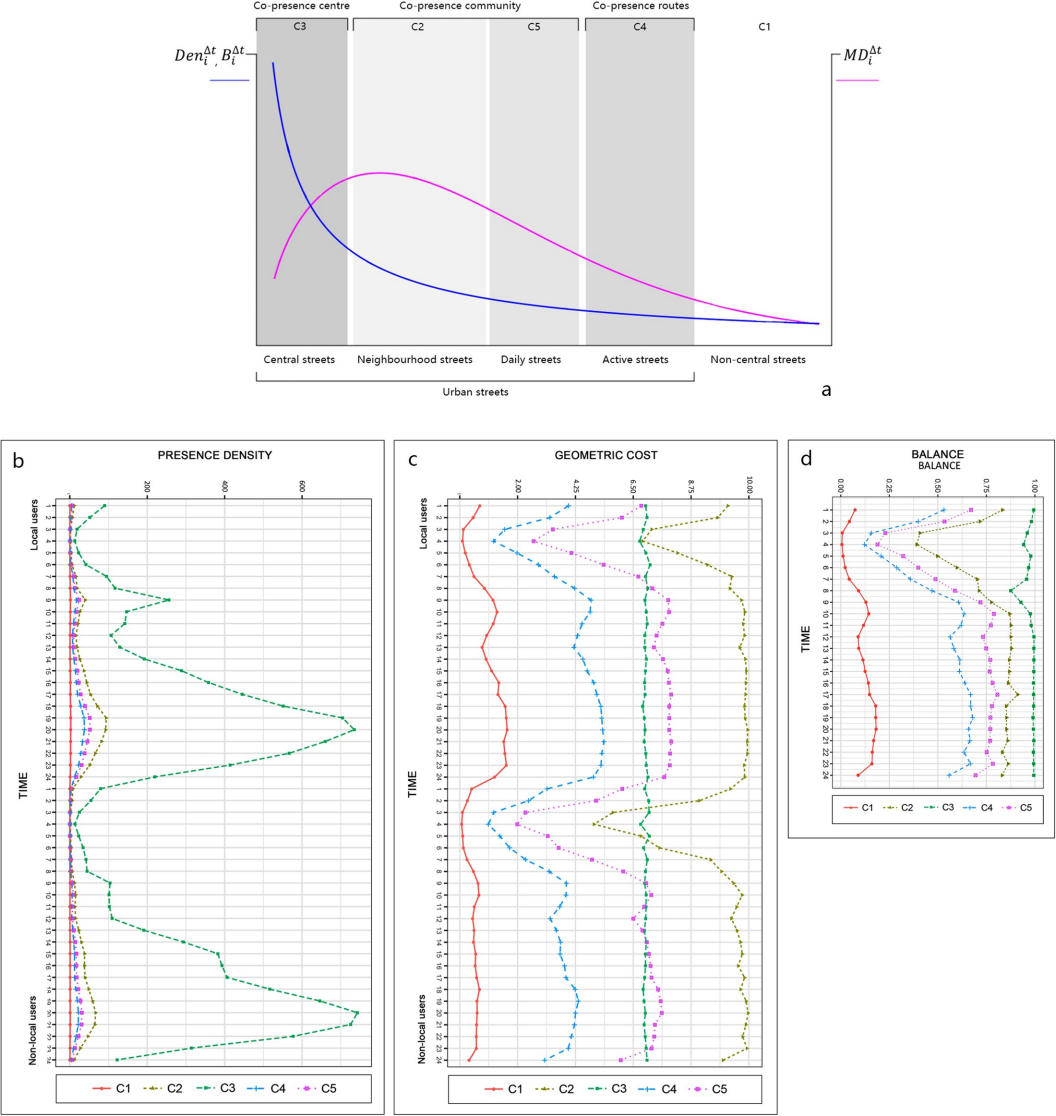
Street typology in terms of co-presence modes. ((Annotation of streets (a), mean values of the physical presence density (b), cognitive cost (c), and balance degree (d) across time for local and non-local users in each cluster in Central Shanghai).

Fig 13 illustrates the results of street typology in terms of the co-presence modes. It provides a new perspective on how street hierarchy is re-characterized by people’s interaction activities. There are five types of streets that are clustered and defined based on the statistical performance of the inter-group difference and within-group similarity. These groups are annotated according to the mean values of the co-presence density and distance for each group of space users (Fig 14). The first detected group of streets (C3) is named “central streets” and has the highest degree of co-presence density and balance and a middle length of cognitive distance. These streets are more akin to a patchwork, representing core areas for citizens to encounter one another. These areas include various types of centers, including historic cores covering the Nanjing Road area and Jing’an District, which were historically colonial areas, newly planned urban centers, such as the Lujiazui central business district, Wujiaochang, Xujiahui, and areas near large urban complexes, such as the Hongqiao transport hub and Hongkou Stadium. It is evidence that places for human face-to-face interactions in contemporary society are not only public spaces but also the public areas where large complexes for experiential consumption are clustered. The second and third groups of streets maintain high levels of co-presence intensity, but they can clearly be distinguished from one another according to their detailed properties in temporal co-presence density and distance. The second group of streets (C4) is called “active roads” because the streets are highlighted on the basis of the low degrees of co-presence density and cognitive distance. In these streets, people are more likely to see one another when they are present simultaneously, although there are a few people clusters present. The third group of streets (C2) is “daily streets” where co-presence density and mean angular distance are both high. When passing through these streets, people must make more geometric turns than those on “active roads” even though the flow volume is greater. Another group of streets is the “neighborhood streets.” These are the places where many people gather, but their face-to-face probability is largely constrained by the spatial configuration. The average angular step depth for people to encounter one another in certain streets within this group is more than nine for most of a typical workday. There are such places in historic areas and central areas in large residential communities. As an example, the traditional Chinese inner city of Shanghai is defined in this group due to its complexity in the organization of space. The remaining group is defined as “non-central streets” where few people will be present even if the cognitive distance for their encounter is short.

The change of the average balance degree between the local and non-local users is recorded in Fig 14d. The ranking of street groups in the balance level is the same as the order in the presence density. This can be considered as proof that the volume of urban flows is a fundamental factor impacting the physical co-presence between the different groups of people defined in this study. However, the dynamic change of the balance index values shows that it is not merely a dependent variable for the presence density. Central streets retain the highest mean values over 24 hours with little variation, whereas neighborhood streets, daily streets, and active roads are characterized by three similar trends with higher co-presence balance values after 10 am, and the lowest values at 3 am. This consistency demonstrates that dynamic shifts of co-presence patterns in most urban streets are inherently significant except for in the detected central streets. The non-central streets, as expected, are inferred to have the lowest degree of co-presence balance.

The spatial patterns of the clustered streets also reveal the potential relationship between co-presence modes and street hierarchy. The “central streets” (C3, *red*) tend to be found in urban cores, and are geometrically connected by the “active roads” (C4, *orange*) which are typically the main roads in the central area. The “daily streets” (C5, *magenta*) are typically the paths interlinking the peripheries of the blocks defined by the active roads, while the “neighborhood streets” (C2, *cyan*), are largely private paths encompassed by daily streets. They form the ‘co-presence communities’ where the local living agglomerations are captured. In contrast, “non-central streets” (C1, *light pink*) typically form the backgrounds of the other urban roads, which are relatively non-urban and mainly include expressways or country roads that serve non-pedestrian travelling purposes. The relevant visualization can also be interpreted as a result of urban communities subdivided by active roads and identified by the modes of co-presence between local walkers and non-local visitors. These results yield that the social hierarchy of urban spaces is characterized by the ways in which people encounter others configurationally.

### Physical co-presence intensity as a dynamic network centrality

**Fig 15:**
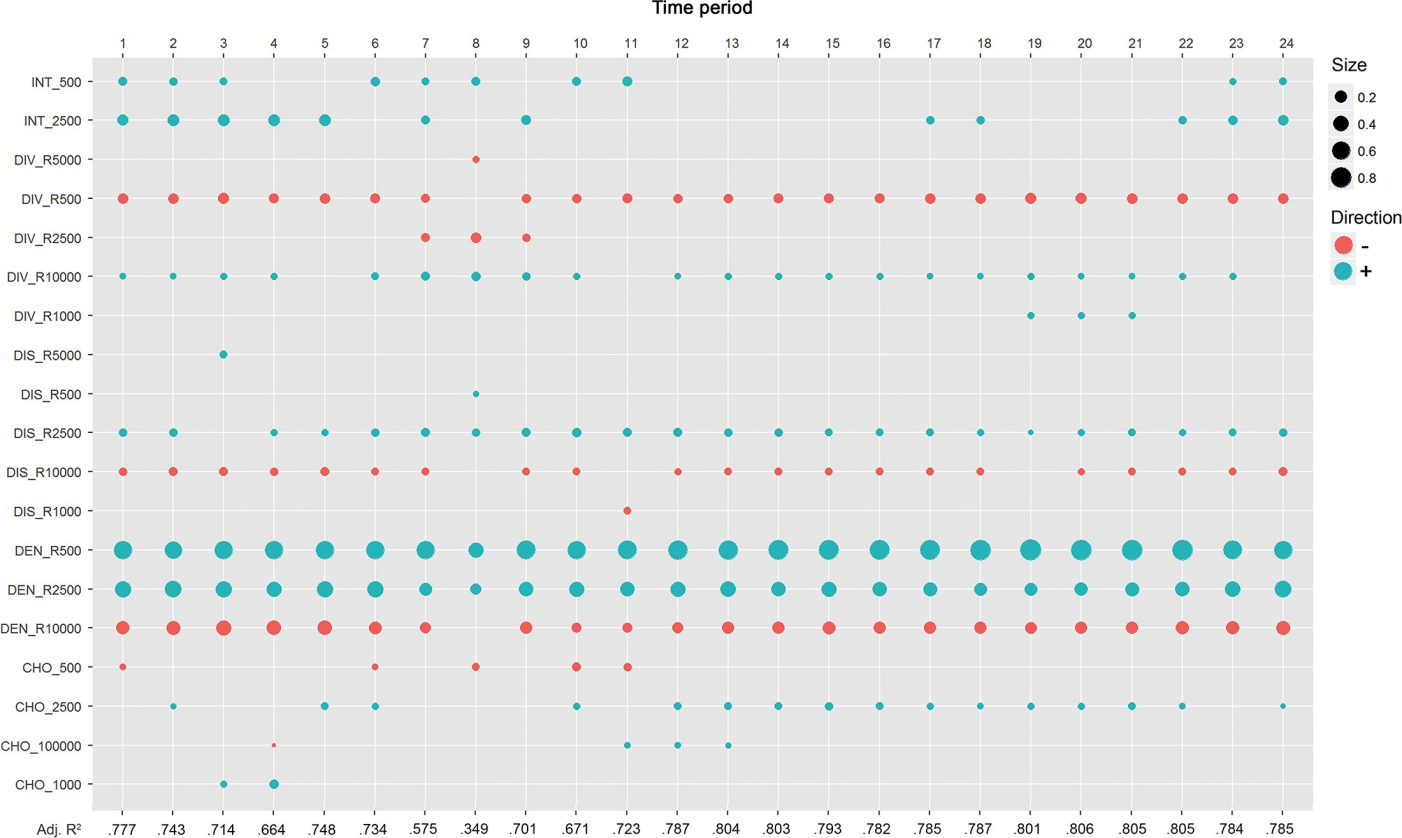
Coefficients of the significant centrality variables in the models predicting physical co-presence intensity patterns.

The introduced physical co-presence intensity patterns uncover the dynamic centrality structures of cities in every street. In order to inspect how these structures differ from those inferred by the existing static network centrality distributions, we use regression technologies to explore their interrelationships. The results of the stepwise multivariate regression method are documented in Fig 15. It is standard for all model specifications that accessible function densities at the micro and mesoscales are the main determinants significantly correlated with physical co-presence intensity. The global density, however, exerts negative effects on it. This suggests that land-use clustering at smaller scales provides the basic landscape for physical interaction between local and non-local citizens. Another general documented trend is that pedestrian land-use diversity is a suppressed factor but global diversity is an augmented factor, which yields a tendency where the local mixture of urban functions is not simultaneously preferred by the two defined groups of people if the density effects are fixed. Likewise, the places where people are more likely to be co-present are inferred by a longer angular distance at the local scale, but less cognitive efforts are found at the global scale. These results imply that co-presence occurs at the locations that are metrically proximal to, but configurationally distanced from, the areas where the clustering of urban functions manifest at the middle scale. In other words, the stages for the physical co-presence may not only be high streets, but are also likely to be the places connected to central streets as the interfaces between one center and another.

Angular integration and choice variables at low levels are positively associated with the change of co-presence intensity across time, but their statistical significance varies. Angular integration variables are more significant in the specific models before 12 am. In the afternoon, angular choice variables are more significant than the integration elements. This may indicate the fact that the co-presence that occurs in the morning is related to the to-movements driven by the closeness between spaces. This demonstrates that developing areas—places lacking sufficient local amenities but that are fulfilled with adequate housing and employment opportunities—are captured by integration variables at local scales and play more important roles in the spatial co-presence patterns in the morning work hours and late at night when people are commuting across the city to their workplaces and homes. By contrast, non-working and non-residential activities are more dominant during other periods within a typical workday; therefore, the impact of angular integration becomes less statistically significant. In other words, spatial centrality measures are significant factors for predicting temporally shifting co-presence patterns using functional centrality indices, and the dynamic change of their significance reveals the composition of various types of urban movements across time.

When the goodness-of-fit for every model is scrutinized, the physical co-presence patterns in the streets are proven to be properly captured by the centralities of the spatio-functional context where they are embedded, which suggests that the physical co-presence intensity index can reflect the spatio-functional interactions. This is also evident that spatial network and function patterns constitute the fundamental fabrics of human dynamics and interactions [52]. For most of a typical workday, the models with both families of configurational centralities maintain correlation coefficients greater than 0.65. However, this trend is interrupted during the period around the morning peak from 6 am to 8 am. This result implies that the co-presence patterns may be simultaneously driven by other variables that are currently absent from the present models in a more complex sense. The overall performance of these models specified according to the time period in question suggests a complimentary relationship between urban forms and functions for an in-depth understanding of people’s interactions in the streets. These results further exhibit that the physical co-presence that occurs in an urban reality is more complex than was expected and hypothesized in the traditional theory of space syntax where urban space is considered as a regulator of land-use distributions. In comparison to the roles that the spatial grid plays, the effects of the geometric properties of land-use patterns on spatiotemporal encounters are more direct and powerful.

An investigation on the relationship between the co-presence mode and network centralities is conducted through a multinomial logistic regression, in which the set of daily streets is used as the reference group. The related results are shown in Table 2, in which the outcomes of the likelihood ratio (LR) Chi-Square test and the pseudo R2 estimation show that the proposed model has good explanatory power. Although the signs of one type of centrality measure at different scales may vary, their coefficient sizes still create a clear picture regarding the structure of determinants. Streets with greater accessible function densities at various scales are more likely to be the central streets and neighborhood streets; those with less land-use density are defined as active roads or non-central streets. Moreover, the function density, accessible function diversity, and mean angular distance are also significant factors. With the increase of the average angular distance to the nearby functions and the decline of the function diversity at 1,000 m, the probability of the streets being categorized in the group of non-central streets relative to the risk of falling in the referent group grows. The two most significant factors that distinguish neighborhood streets from daily streets are the mean angular distance and the function diversity at pedestrian levels. Streets that are more configurationally close to the reachable functions with a higher mixture degree at 1,000 m are more likely to be defined as neighborhood streets. This evidence suggests that neighborhood streets facilitate local functions in residential communities, which are hidden geometrically from the places where face-to-face encounters between locals and non-locals occur frequently.

**Table 2:** Results of the multinomial logistic regression for the impact of centrality structures on the co-presence typology of streets.

Intercept	C1: Non-central streets		C2: Neighborhood streets		C3: Central streets		C4: Active roads	
	*B*	exp(*B*)	*B*	exp(*B*)	*B*	exp(*B*)	*B*	exp(*B*)
	2.835***		-10.093***		-97.526***		3.788***	
Urban function connectivity measures								
DEN_R500	-2.411***	0.090	0.934***	2.544	7.748***	2315.279	-1.002***	0.367
DEN_R2500	-4.552***	0.011	1.657***	5.243	10.024***	2.255*10^4^	-1.517***	0.219
DEN_R10000	-0.299***	0.742					-0.273***	0.761
DIV_R500	0.235***	1.265	0.231***	1.260	-5.121***	0.006	-0.104**	0.586
DIV_R1000	-1.729***	0.177	1.356***	3.880	10.893***	5.379*10^4^	-0.534***	1.166
DIV_R2500	0.720***	2.054					0.154***	1.659
DIV_R5000	0.616***	1.852	-0.412***	0.662	-23.082***	0.000	0.506***	0.042
DIV_R10000	-0.136*	0.873			127.022***	1.461*10^6^		
DIS_R500	-0.303***	0.739	0.887***	2.427			-0.336***	0.715
DIS_R1000	7.419***	1,666.953	-9.173***	0.000	-4.682***	0.009	5.219***	184.657
DIS_R2500	-0.295***	0.745			2.715***	15.108		
DIS_R5000					-1.968***	0.140		
DIS_R10000					1.184***	3.268		
Space syntax metrics								
INT_500	-0.376***	0.687	0.235***	1.265			-0.099***	0.905
INT_2500	0.274***	1.361	-0.218***	0.614	1.114***	3.046	0.168***	1.183
CHO_500	-0.236***	0.790			-0.585***	0.557	-0.225***	0.798
CHO_2500	-0.746***	0.474					-0.459***	0.632
CHO_100000	0.999***	2.714					0.674***	1.961
Chi-Square = 160,023 Sig. = 0.000								
Pseudo *R*^2^(Cox and Snell) = 0.823								

*, significant at *p* < 0.1;

**, significant at *p* < 0.05;

***, significant at *p* < 0.01. Reference group: daily streets (C5). *B*, Estimated coefficient; exp(*B*), odds ratio for that coefficient.

Compared to the referent group, central streets obtain a higher probability of having a higher degree of land-use mixture at the local and global scales with a shorter cognitive distance to them. The impact of the space syntax centrality indices is less significant than the effects of the urban function connectivity measures on segmenting co-presence styles in the streets. The most significant factor in the spatial centrality measures is the angular integration at 2,500 m, which is positively related to the probability that the streets in question are central streets. On the whole, given that the impacts of other factors are constant, the urban function connectivity measures at the pedestrian level, the angular integration at the mesoscale, and the angular choice at the macroscale in the family of space syntax centrality metrics are critical factors for differentiating between the street-based co-presence modes.

## Discussion

This exploratory research examines approaches to using social media check-in records to study the face-to-face interaction potentials in Central Shanghai. To do this, we develop a variety of metrics on the basis of the concept of physical co-presence intensity. The patterns of these metrics provide dynamic snapshots of centrality structures within the urban street network. This highlighted the importance of the urban built form in connecting people geometrically. The findings and methods presented here have implications for the study and planning of urban environments in a modern, digitized society.

This study delivers a PST model in which the capitals for people to interact include three principal dimensions, the social difference between people, the spatial distance (the metric and geometric distance), and the time cost of people’s presence. By giving a time interval and a distance radius, the delivered physical co-presence intensity addresses the interplay among the co-presence density, balance, and cognitive distance in every street and through spatial grids based on individuals’ trajectories extracted from their consecutive check-in records. It portrays the spatiotemporal patterns of urban centrality structures showing the perceived publicness in every street, and enables the production of deeper knowledge on how the offline built environment is used by the online population in the current digitized world, which can be generalized to all real space users.

Co-presence potential patterns of Central Shanghai (μ = 0.5; Δt = 1h; r = 750m) over time reveal their temporal complexity. Developed city centers as well as other planned centers and streets around large-scale social complexes, such as transport hubs and shopping malls, are crucial spaces for people to encounter one another. Our regression results suggest that the physical co-presence between local and non-local users does not always occur in high streets as expected, but in the streets connecting these centers where the pedestrian land-use mixture is lower and the angular distance to the land-uses is longer. The impact of the angular integration in the regression models for describing the deviation of co-presence patterns is more significant in the morning when developing areas are more likely to be destinations during commuting times.

Five co-presence modes are discovered in the streets via a data-driven process, showing the social translation of a street hierarchy to a socially functional hierarchy for physical interactions. They are then annotated as central streets, active roads, daily streets, neighborhood streets, and non-central streets in accordance to the temporal presence patterns of the defined social groups. Central streets are akin to a patchwork, indicating a polycentric structure of face-to-face co-presence. The patterns of other detected groups of streets, on the other hand, show a relatively hierarchal structure. The results of multinomial logistic regression indicate that the pedestrian functional centralities are the main factors that discriminate the detected clusters, and the spatial centralities at the larger scales are also significant elements, though their impact is less than that of the functional indices.

The impact of this work is potentially wide-ranging. First, it is shown that a social media dataset can be used to understand face-to-face interaction potentials perceived in the real public space, with large coverage and at a fine spatial resolution. Second, we propose a street-based framework to quantify the spatiotemporal co-presence between defined groups of people. This framework is flexible and can be extended to measure other co-presence phenomena. For instance, we can use other definitions of social groups to explore the physical co-presence potentials between them. We can also modify Eq 7 to $I_{i}^{\Delta t}=\frac{{{Den}_{i}^{\Delta t}}^{{(1-B}_{i}^{\Delta t})}}{{MD}_{i}^{\Delta t}},{\{d}_{ij}\leq r\}$, and the resulting index can be considered as a new form of dynamic segregation, highlighting the largest potentials dominated by certain group(s). Third, we introduce the measure of co-presence intensity which could be considered a spatiotemporal centrality index across places. It extends the current space syntax model, which purely focusses on the static spatial networks, to a more comprehensive, dynamic model of urban centrality, with land-use, spatial networks, and temporal and individual components, thereby encompassing the four recognized domains of accessibility measurements [53]. This could facilitate further discussion on the ways in which trajectory data can contribute to configurational analysis. Finally, the delivered framework can be used as a planning-support-tool based on social media data in the planning and design process, in which planners can test the changes to the temporal co-presence intensity in the streets by re-organizing the spatial layouts until a satisfying result emerges.

Further research can be conducted on, but is not limited to, the following areas. The time interval for computing the co-presence intensity in this study was set at one hour in order to avoid the bias caused by the scarceness of the data. Future research with larger data-sets could investigate the use of shorter time intervals to produce results with better temporal resolution. Furthermore, individual check-in trajectories could be enriched by combining these data with other formats of big data resources, such as cell phone location information, to avoid the scarcity of social media data and to calibrate a better model that describes and distinguishes users’ social groups more properly. Moreover, only street networks, a horizontal urban system, are focused on in this article, thus neglecting the vertical and in-door co-presence which might not always be consistent with urban reality. Thus, the following efforts should be made to properly address the physical interactions within large complexes. This can be done by applying the proposed method based on the network representation of the in-door built environment.

## Supporting information

S1 Appendix Algorithm for computing physical co-presence intensity. (DOCX)
