# Supplementary material for: Physical co-presence intensity: Measuring dynamic face-to-face interaction potential in public space using social media check-in records
Source: PLoS One. 2019 Feb 11;14(2):e0212004. doi: 10.1371/journal.pone.0212004 (PMC6370218; doi:10.1371/journal.pone.0212004)
Supplement: S1 Appendix — (DOCX) [file pone.0212004.s001.docx]

**S1 Appendix. Algorithm for computing physical co-presence intensity**

This appendix is to supplement the description of the data processing introduced in Section 03 of the method. The algorithmic function and flow chart shown below provide a very detailed description about the computational processes. Interested readers are encouraged to use any platform they like to achieve the same aims.

**Input**: Street segment-based shape file; Point-based trajectory shape file

**Output**: Street segment-based shape file with new fields of the delivered indices

**While** (not last segment) **do**

Start with the midpoint of the first segment (i) in the attribute table of streets

**If** (the current segment is processed) **then**

Exit

**End**

Assign every check-in point in trajectory data to its nearest street segment

Calculate the duration of all check-in points and divide it by the fixed time interval $\Delta t$

Create a dual graph of street network ($G(V,E)$) using the midpoints of segments as vertices (V) and the conjunctions as edges (E)

Calculate the values of angular change in all edges (E)

Calculate the minimums network distance between midpoint (i) and check-in points (j) along the shortest path

Calculate the minimums angular distance between midpoint (i) and check-in points (j) along the least angular change path

**If** (the minimums network distance $>$ threshold r) **then**

Let the weight of selected check-in points be 0

**If** (the check-ined time$\notin\Delta t$) **then**

Let the weight of the selected check-in points be 0

Sum the weights of all check-in points, namely W_ALL

**For** (UserID $\in$ the list of UserID) **do**

Calculate the cumulated weights (UserW) for every user with a UserID

Calculate the number of check-ins (Nche) with non-zero weights for every user

**If** ($UserW < threshold \mu$) or (Nche =1) **do**

Classify the selected users as the non-locals

**Else**

Classify the selected users as the locals

**End**

**If** (UserID $\in$ the list of UserID for the non-locals) **do**

Sum the weights of all check-in points for the non-locals, namely W_NL

Sum the minimums angular distance between the midpoint (i) and the check-in points for the non-locals, namely A_NL

Calculate the mean minimums angular distance between the midpoint (i) and the check-in points for the non-locals, namely Av_NL

Calculate presence intensity for the non-locals, namely PI_NL

**Else if** (UserID $\in$ the list of UserID for the locals) **do**

Sum the weights of all check-in points for the locals, namely W_L

Sum the minimums angular distance between the midpoint (i) and the check-in points for the locals, namely A_L

Calculate the mean minimums angular distance between the midpoint (i) and the check-in points for the locals, namely Av_L

Calculate presence intensity for the locals, namely PI_L

**End**

Normalise the physical presence intensity for the local and non-local people

Calculate the entropy based on W_NL and W_L, namely E_ALL

Sum the minimums angular distance between the midpoint (i) and the check-in points for the non-locals, namely A_ALL

Calculate the mean minimums angular distance between the midpoint (i) and the check-in points for the non-locals, namely Av_ALL

Calculate the physical co-presence intensity, namely PCI

**End**

**End**

Pick out another unprocessed segment to continue

**End while**
